# Body Adiposity Index Performance in Estimating Body Fat Percentage in Colombian College Students: Findings from the FUPRECOL—Adults Study

**DOI:** 10.3390/nu9010040

**Published:** 2017-01-17

**Authors:** Robinson Ramírez-Vélez, Jorge Enrique Correa-Bautista, Katherine González-Ruíz, Andrés Vivas, Héctor Reynaldo Triana-Reina, Javier Martínez-Torres, Daniel Humberto Prieto-Benavides, Hugo Alejandro Carrillo, Jeison Alexander Ramos-Sepúlveda, Emilio Villa-González, Antonio García-Hermoso

**Affiliations:** 1 Centro de Estudios para la Medición de la Actividad Física CEMA, Escuela de Medicina y Ciencias de la Salud, Universidad del Rosario, Bogotá 111221, Colombia; jorge.correa@urosario.edu.co (J.E.C.-B.); danielprietob@gmail.com (D.H.P.-B.); 2 Grupo de Ejercicio Físico y Deportes, Vicerrectoría de Investigaciones, Universidad Manuela Beltrán, Bogotá 110231, Colombia; katherine.gonzalez@docentes.umb.edu.co (K.G.-R.); jose.vivas@docentes.umb.edu.co (A.V.); 3 Grupo GICAEDS, Facultad de Cultura Física, Deporte y Recreación, Universidad Santo Tomás, Bogotá 110311, Colombia; hectortriana@usantotomas.edu.co (H.R.T.-R.); javiermartinezt@usantotomas.edu.co (J.M.-T.); 4 Grupo GRINDER, Programa de Educación Física y Deportes, Universidad del Valle, Santiago de Cali 76001, Colombia; hugo.carrillo@correounivalle.edu.co; 5 Facultad de Educación a Distancia y Virtual, Institución Universitaria Antonio José Camacho, Santiago de Cali 760045, Colombia; jalexanderramos@admon.uniajec.edu.co; 6 Department of Education Sciences, University of Almería, Almería 04120, Spain; evilla@unach.edu.ec; 7 PROFITH “PROmoting FITness and Health through Physical Activity” Research Group, Department of Physical Education and Sport, School of Sport Sciences, University of Granada, Granada 18010, Spain; 8 Laboratorio de Ciencias de la Actividad Física, el Deporte y la Salud, Universidad de Santiago de Chile, USACH, Santiago 7500618, Chile; antonio.garcia.h@usach.cl

**Keywords:** body composition, validity, students, body adiposity

## Abstract

Recently, a body adiposity index (BAI = (hip circumference)/((height)(1.5))^−18^) was developed and validated in adult populations. The aim of this study was to evaluate the performance of BAI in estimating percentage body fat (BF%) in a sample of Colombian collegiate young adults. The participants were comprised of 903 volunteers (52% females, mean age = 21.4 years ± 3.3). We used the Lin’s concordance correlation coefficient, linear regression, Bland–Altman’s agreement analysis, concordance correlation coefficient (*ρc*) and the coefficient of determination (*R*^2^) between BAI, and BF%; by bioelectrical impedance analysis (BIA)). The correlation between the two methods of estimating BF% was *R*^2^ = 0.384, *p* < 0.001. A paired-sample *t*-test showed a difference between the methods (BIA BF% = 16.2 ± 3.1, BAI BF% = 30.0 ± 5.4%; *p* < 0.001). For BIA, bias value was 6.0 ± 6.2 BF% (95% confidence interval (CI) = −6.0 to 18.2), indicating that the BAI method overestimated BF% relative to the reference method. Lin’s concordance correlation coefficient was poor (*ρc* = 0.014, 95% CI = −0.124 to 0.135; *p* = 0.414). In Colombian college students, there was poor agreement between BAI- and BIA-based estimates of BF%, and so BAI is not accurate in people with low or high body fat percentage levels.

## 1. Introduction

Obesity has reached an epidemic proportion, being the main cause of death and disability around the world [1]. Excess adipose tissue is associated with cardiovascular disease (CVD) risk factors such as hypertension, diabetes mellitus and dyslipidaemia [2,3,4]. National trends in CVD risk factors show that although there have been marginal improvements in all weight groups, risk factors continue to be higher in obese and overweight subjects [5,6].

A simple and effective measure of adiposity is needed to enable us to estimate the magnitude of the problem and development appropriate management and preventive strategies. Various methods can be used, such as magnetic resonance imaging (MRI), computed tomography, dual-energy X-ray absorption (DEXA), isotopic measurement of body water, whole body plethysmography, bioelectrical impedance analysis (BIA) and underwater weighing [6]. Although these methods of assessing adiposity are accurate, non-invasive, rapid and reliable, they are not routinely used in clinical practice because of their cost. Anthropometric methods of assessing body composition based on measurements of weight, height and body circumference have been used as an alternative to laboratory methods [7]. All these indicators are simple, inexpensive, non-invasive methods that have been validated for use in clinical practice and epidemiological research [8]. In 2011, a new anthropometric indicator was proposed, the Body Adiposity Index (BAI). The BAI is derived from hip circumference and height and was intended to be a direct validated method of estimating body fat percentage (BF%), which was developed in a sample of Mexican Americans and validated in African-American adults [9]. However, validation studies done in populations of various ethnicities have consistently indicated that the BAI tends to overestimate adiposity at lower BF%, and underestimate adiposity at higher BF% [10,11,12,13]. Specifically, BAI does not provide valid estimates of BF% in Caucasian, European or European-American adults [14,15,16,17].

Validation studies in Costa Rica [18] and Brazil [19], based on 199 college students (mean age 18.6 ± 2.4 years) and 706 individuals (mean age 37.3 years ± 12.1), respectively, showed that BAI cannot be recommended as a predictor of BF% in these Latin-American populations [20]. Given the risk of over-nutrition in developing countries, it is necessary to measure its prevalence in vulnerable populations, such as Latin-American adults, in order to identify high-risk groups and develop preventive interventions [7]. At present, there are few global reports on the prevalence of overweight individuals and obesity for low- to middle-income countries experiencing rapid nutrition transitions, such as those in Latin America and Africa [7], although assessment of body composition is of crucial importance in these countries because of the relatively high prevalence of both underweight and overweight [21,22,23,24].

Since the index was developed in samples of Mexican-American and African-American individuals, the effectiveness of BAI as an alternative method of estimating BF% and the validity of BAI as a predictor of risk of cardiovascular disease in other ethnicities needs further investigation. As far as we know, our study is the first to analyze the validity of BAI to use as an alternative measure for BMI in Colombian collegiate students in a large cohort and in both genders. The aim of this study was to evaluate the performance of BAI in estimating BF% in a sample of Colombian college students, with BIA used as the reference method.

## 2. Methods

### 2.1. Participants

We implemented the cross-sectional component of the FUPRECOL study (Association between Muscular Strength and Metabolic Risk Factors in Colombia) in Bogota, Colombia, during the 2013–2014 college year [25,26]. We recruited a convenience sample consisting of 903 volunteers (51.9% females, mean age = 21.4 years ± 3.3; range 18–35) who were of low-to-middle socioeconomic status (SES) (i.e., in classes 1 to 4 of the six-class scheme defined by the Colombian government) and were enrolled in public or private university in the capital district of Bogota and Cali, Colombia. Students were informed that participation was voluntary and that there was no penalty for not participating. Inclusion criteria were: no self-reported history of inflammatory joint disease or neurological disorder; not an elite athlete. Participants were not compensated. Exclusion criteria were: a medical or clinical diagnosis of a major systemic disease (including malignant conditions such as cancer); type 1 or 2 diabetes; high blood pressure; hypothyroidism or hyperthyroidism; a history of drug or alcohol abuse; regular use of multivitamins; inflammation related to trauma or contusions; infectious conditions; BMI (body mass index: weight in kg/height in m^2^) ≥ 35. The institutional ethics committee approved the study (Universidad Manuela Beltrán No. 01-1802-2013) in accordance with the latest version of the Declaration of Helsinki. After providing written, informed consent to participation, volunteers were given an appointment for a testing session at the University laboratories.

### 2.2. Procedures

Each participant was asked to complete a health questionnaire and we also collected sociodemographic data and information about personal and family pathological background. After completing the questionnaire, participants were instructed to change into shorts and a t-shirt and remove any metal or jewelry from their persons. Anthropometric variables were assessed by a nutritionist in accordance with the International Society for the Advancement of Kinanthropometry guidelines [27]. Data were collected in the morning, in a single session after a fast of approximately 12 h, by a single trained and experienced evaluator. Body weight was measured with participants barefoot in their underwear, using electronic scales (Model Tanita^®^ BC 420MA, Tokyo, Japan). Height was measured using a mechanical stadiometer platform (Seca^®^ 274, Hamburg, Germany). We calculated BMI from the height and weight measurements. Weight status was determined according to World Health Organization (WHO) criteria for obesity (BMI ≥ 30) and overweight (BMI ≥ 25) [28]. Waist circumference (WC; in cm) was measured using a tape measure, at the smallest point between the lower costal border and the iliac crest; where this was not evident, it was measured at the midpoint between the last rib and the iliac crest (Ohaus^®^ 8004-MA, Parsippany, NJ, USA). Hip circumference (in cm) was measured at the largest point around the buttocks with the tape horizontal and parallel to the ground using a tape with 0.1 mm accuracy (Ohaus^®^ 8004-MA, Parsippany, NJ, USA). A tetrapolar whole body impedance meter (Model Tanita^®^ BC 420MA, Tokyo, Japan) was used to perform the analysis of BF%, similar to previous studies [7,8]. Measurements were made with the participant in a standing position with arms and legs lying parallel to the trunk and separated, so that the thighs were not touching. Before testing, participants were required to adhere to these BIA manufacturer’s instructions [29]: (i) to not eat or drink within 4 h of the test; (ii) to not consume caffeine or alcohol within 12 h of the test; (iii) to not take diuretics within 7 days of the test; (iv) to not do physical exercise within 12 h of the test, and; (v) to urinate within 30 min of the test. An electrical current of 50 kHz was passed through the participant and resistance and reactance were measured. To ensure data quality, the equipment was calibrated daily using a known calibration standard, in accordance with the manufacturer’s instructions [29]. BAI was calculated from hip circumference and height as follow: BAI (BF%) = (hip circumference [cm]/height [m]1.5)^−18^ [9]. We also calculated the waist-to-hip ratio (WHtR).

### 2.3. Statistical Analysis

Statistical analyses were performed using Statistical Package for the Social Sciences software for Windows version 21.0 (IBM Corporation, New York, NY, USA). The Kolmogorov–Smirnov test was used to assess the distributions of variables; *p*-values < 0.05 were considered significant. Statistical analysis consisted of a description of the variables (mean, standard deviation (SD)) and *t*-tests or Chi Square test to check for differences in means or proportions, respectively. The BIA method was treated as the gold standard method. We used separate paired-sample *t*-tests to assess differences between the two methods of estimating BF% for each gender, level of adiposity and weight status. Lin’s concordance correlation coefficient was used to assess the concordance between BAI and BIA separately for males and females [30]. The methods used to assess the relationships between %BF_BIA_ and %BF_BAI_ stratified by gender at different stages were the Bland-Altman analysis, multiple regression analysis and coefficient of determination (*R*^2^) [31].

## 3. Results

Descriptive statistics and between-gender comparisons are shown in Table 1. All of the anthropometric variables, except hip, BMI and BMI ≥ 30 (kg/m^2^), were different in males than in females (*p* < 0.001).

In both males and females, Lin’s concordance correlation coefficient for the association between BF%_BAI_ and BF%_BIA_ was poor, *ρc* = 0.021 (95% CI = −0.174 to 0.184; *p* = 0.408) and *ρc* = 0.029 (95% CI = −0.174 to 0.196; *p* = 0.381), respectively. Males and females were then grouped according to BF% and, as shown in Table 2, BAI underestimated BF% at all levels of adiposity and weight status. In females, a paired-samples *t*-test revealed a difference between the two methods of estimating BF% (difference in means = −3.1 (CI 95% −3.7 to −2.6); BAI 30.0 [5.4]% vs. BIA 26.8 [7.2]% *p* < 0.001). In males, a paired-samples *t*-test revealed a difference between the two methods of estimating BF% (difference in means = −8.7 (CI 95% −9.3 to −8.1); BAI 24.8 [5.5]% vs. BIA 16.0 [6.7]%, *p* < 0.001). Significant differences were found in both genders in students with BMI greater than 25 (*p* < 0.01).

**Figure 1:**
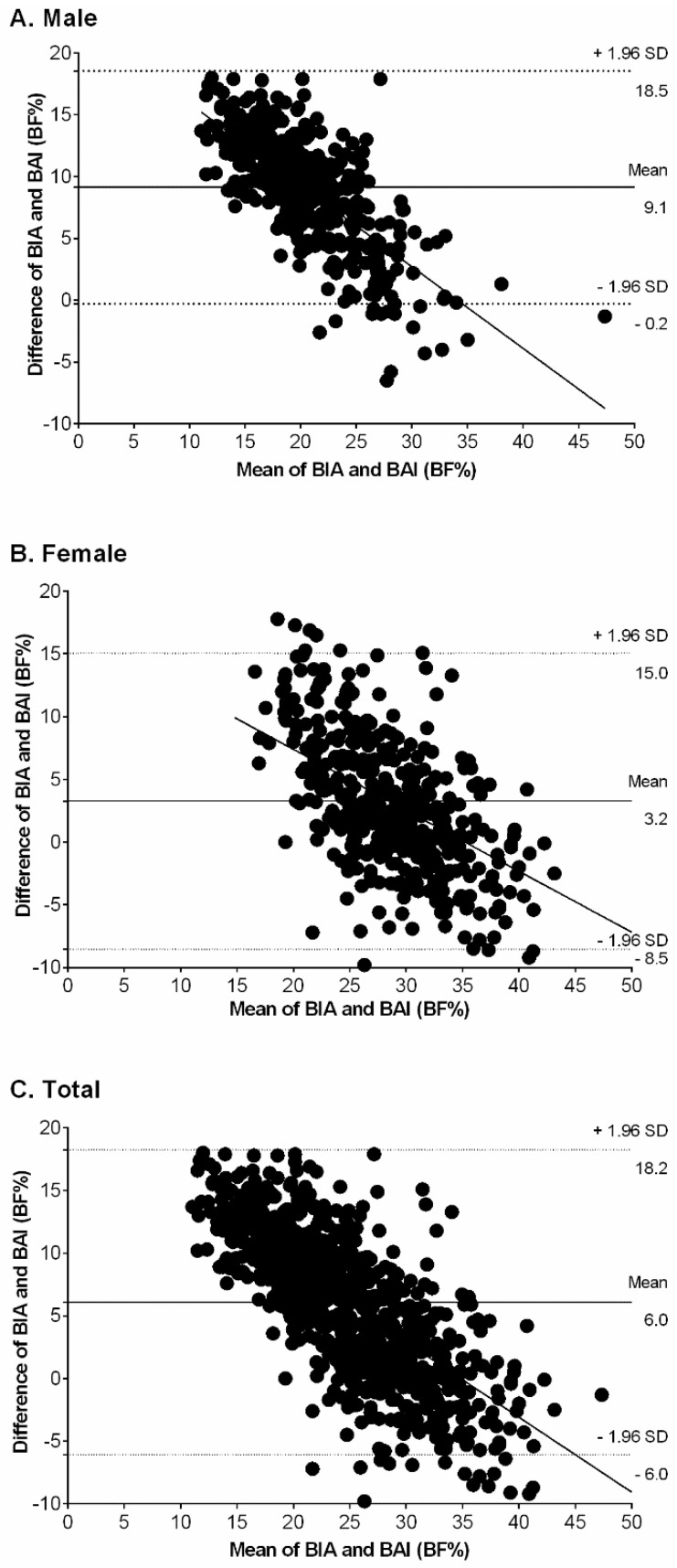
Bland–Altman plots with mean and 95% limits of agreement for comparing BF%_BAI_ and BF%_BIA_ among males (**A**), females (**B**), and total (**C**). The central line represents the mean bias between BF%_BAI_ and BF%_BIA_; the outer lines represent 95% limits.

The Bland–Altman plot (Figure 1) showed that BAI overestimated BF% relative to BIA in males (Figure 1A), females (Figure 1B) and the combined sample (Figure 1C). In men, the bias of the BAI was 9.1 (SD 4.8) BF% (95% CI = −0.2 to 18.5). In women, the bias of the BAI was 3.2 (SD 6.0) BF% (95% CI = −8.5 to −15.0). In the combined sample, the bias of the BAI was 6.0 (SD 4.3) BF% (95% CI = −6.0 to 18.2), indicating that the BAI method significantly overestimated BF% relative to the BIA method. The slopes in Figure 1 show that the correlation between the differences in BAI and BIA, as well as the mean BF% measured using both methods, was higher in females (r = 0.530, *p* < 0.001) than in males (r = 0.461, *p* < 0.001).

**Figure 2:**
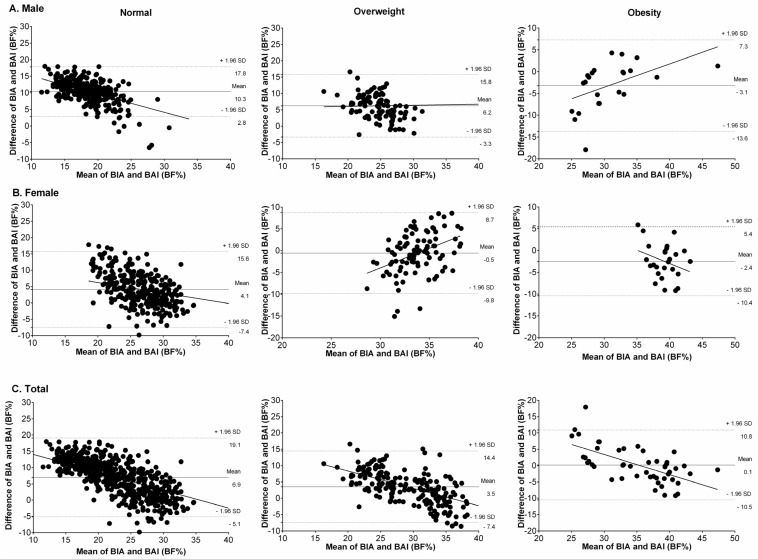
Bland–Altman plots with mean and 95% limits of agreement for comparing BF%_BAI_ and BF%_BIA_ among males (**A**), females (**B**), and total (**C**) according to weight status (normoweight, overweight and obesity). The central line represents the mean bias between BF%_BAI_ and BF%_BIA_; the outer lines represent 95% limits.

Brand-Altman plots stratified by gender and weight status showed that, in individuals of normal weight (Figure 2), the BAI overestimated BF% relative to BIA in males (Figure 2A), females (Figure 2B), and the whole sample (Figure 2C). Bland–Altman plots for the overweight group (middle panel) showed that the BAI overestimated BF% relative to BIA in men and in the combined sample; however, in the obese group (right panel), the BAI underestimated BF% relative to BIA in both genders.

## 4. Discussion

The purpose of the study was to assess the performance of BAI as an estimator of BF% in a sample of Colombian college students. The main finding was the BAI’s lack of predictive validity as a method of estimating BF% in both genders relative to BIA (bias = 6.0%). The Bland–Altman plots showed that BAI tended to overestimate adiposity in males (bias = 9.1) and females (bias = 3.2) relative to the criterion measure, namely BIA. Another finding was that BAI overestimated BF% in both genders, particularly in participants with a higher level of adiposity and in heavier participants. We concluded, therefore, that BAI does not seem to be appropriate to determine BF% in the Colombian young adult population.

Although it has been suggested [9,32] that BAI can provide an estimate of BF% without further adjustment, our results indicate that these estimates are systematically biased by gender, level of adiposity and weight status. We agree with Freedman et al. [33] that analyses of body fatness that do not control for gender should be treated with caution. As females are generally shorter than males and have more BF%, an analysis of the association between height and BF% would greatly overstate the strength of the association. In our study, overall BAI overestimated BF% by 6.0%, a level of bias that is fairly similar to that reported in 623 European-American adults who participated in the Fels Longitudinal Study [34] and in a study [33] of 1151 adults that was based at the Body Composition Unit of the New York Obesity Nutrition Research Center. We found that in both genders, BAI overestimated BF% by about 3.5%; this may have been due to the fact that our participants tended to be overweight (mean BMI = 26.8 ± 1.4). Due to differences between measurements that vary substantially according to level of adiposity, weight status and gender (Table 3), one would expect observed gender differences to vary across studies according to the participants’ adiposity. Ethnicity is another factor that greatly influences shape and body composition. Earlier studies based on samples of various ethnicities, such as European Americans [9], Mexican Americans [32], African Americans [33] and Latin Americans [10,15,25], showed that the BAI overestimates BF% at lower levels of adiposity. Similar to our study, some other studies have shown that the BAI overestimates BF% at higher levels of adiposity [10,11,12]; however, there are also reports that BAI underestimates BF% at higher levels of adiposity [35,36]. It is difficult to compare the results of this study with those of earlier studies, as the earlier studies used a variety of different measurement devices such as foot-to-foot BIA, devices with adhesive tape and multi-frequency devices.

An assessment of the validity of BAI as an estimator of BF% in severely obese individuals [11] using BIA as the reference method found large individual errors in predictions of BF%. Another study of 19 severely obese, non-diabetic females awaiting bariatric surgery [10] showed that the BAI underestimated BF% by up to 2.2% relative to BIA. In contrast, in a sample of Costa Rican students, BAI under- and over-estimated BF% relative to DEXA in females and males, respectively [18]. Even in a population of young adults, BAI- and BIA-based BF% estimates were only weakly correlated [12]. The findings of these studies, together with our results, suggest that when using the BIA method of measuring BF%, clinicians and exercise scientists should report details of the procedure and equipment used to avoid misinterpretation of findings. Lohman [37] considered an error of 4% in estimates of BF% to be reasonable. This may suggest the need to validate field methods commonly used in this environment with other laboratory tests. The reasons for the discrepancy between BAI and BIA are not clear, but as the BAI quantifies adiposity based on height-adjusted hip circumference, differences in the distribution of body fat may be reflected in BAI [38]. Regarding the difference between genders, females have higher levels of body fat than males and their BF% is differently distributed [25], whilst males tend to be taller [25]. Ethnic differences in anthropometric profile and body composition can change the relationship between anthropometric measurements and BF%, meaning that an equation derived from research in one population is invalid in other populations. In addition, weaker associations have been reported between cardiovascular risk factors and BF% by BAI than with WC, WhtR, and BMI [25,39].

Our study has several important limitations. Cross-sectional design limits causal inferences. We have used the BIA as the “gold standard” for adiposity and not the DEXA method. Furthermore, factors that may affect the results are water retention, use of diuretics, hydration status, menstrual cycle, level of BF% and ethnicity, which may also represent a limitation of the use of BIA [40]. Overall, BIA is a useful tool for clinical studies and large epidemiological studies with diverse populations, particularly in Latin-American nations; however, for individual assessment of fat mass, BIA has limited use [41,42]. Therefore, the results that we found must be verified in other age classes and for BMI groups higher than 35 kg/m^2^. Finally, we have not considered the potential impact of recognized determinants, such as socioeconomic status, metabolic biomarkers, physical activity patterns, and physical fitness, which modulate growth and levels of adiposity. The strengths of our study include a large sample size and an equal ratio of males to females.

## 5. Conclusions

In summary, the BAI is not recommended as a method of estimating BF% in young adults from Colombia. The extrapolation of an equation for estimating BF% based on measurements of hip and height for the Colombian population should be viewed with caution due to Colombian ethnicity being composed of a mixture of Amerindians, Europeans, and Africans, one of the most heterogeneous populations in the world, and conferring their peculiar characteristics. The BAI does not appear to be a good alternative to the usual anthropometric indicators of obesity such as waist or hip circumference, WHtR and BMI. We emphasize the importance of a simple and inexpensive method for adiposity estimation in LMICs where sophisticated equipment is not widely availability. Further epidemiological studies examining the utility of BAI for Latin-American populations are still needed for a better understanding of the validity of this new index.

## Figures and Tables

**Table 1:** Characteristics of study subjects as a whole and by gender.

Characteristics	Women (*n* = 469)	Men (*n* = 434)	Total (*n* = 903)	*p*
Age (years)	21.5 (3.2)	21.3 (3.4)	21.4 (3.3)	0.478
Height (m)	1.60 (0.10)	1.72 (0.07)	1.66 (0.11)	0.001
Weight (kg)	58.9 (10.0)	69.9 (12.5)	64.2 (12.5)	0.001
Waist (cm)	72.0 (8.1)	79.1 (9.8)	75.4 (9.6)	0.001
Hip (cm)	97.0 (8.8)	97.5 (9.5)	97.2 (9.1)	0.480
WHtR	0.45 (0.05)	0.46 (0.06)	0.45 (0.05)	0.035
BF%_BIA_	26.8 (7.2)	16.0 (6.7)	21.6 (8.8)	0.001
BF%_BAI_	30.0 (5.4)	24.8 (5.5)	27.5 (6.0)	0.001
BMI (kg/m^2^)	23.0 (3.7)	23.5 (3.7)	23.2 (3.7)	0.097
BMI ≥ 30 (kg/m^2^)	26 [5.5]	23 [5.2]	49 [5.4] *	0.816

Data are expressed as mean (SD) or *n* [%]. *p*-values are given for comparison between women and men. Significant between-gender differences by *t*-test or chi-square *. BIA = bioelectrical impedance analysis; SD = standard deviation.

**Table 2:** Body fat percentage by BAI and BIA according to different levels of adiposity and weight status by gender.

Characteristics	Female						Male					
	*n*	BF%_BAI_	BF%_BIA_	*p*-Value	Difference between Measures (CI 95%)	*R*^2^	*n*	BF%_BAI_	BF%_BIA_	*p*-Value	Difference between Measures (CI 95%)	*R*^2^
All	469	30.0 (5.4)	26.8 (7.2)	0.001	−3.1 (−3.7 to −2.6)	0.280	434	24.8 (5.5)	16.0 (6.7)	0.001	−8.7 (−9.3 to −8.1)	0.212
Level of adiposity (%) *												
≤20	87	26.5 (2.9)	16.2 (3.1)	0.001	−10.3 (−11.1 to −9.4)	0.041	332	23.8 (3.9)	13.1 (4.1)	0.001	−10.7 (−11.1 to −10.2)	0.283
20–30	235	29.1 (5.7)	25.6 (2.8)	0.001	−3.4 (−4.1 to −2.6)	0.046	88	27.7 (6.4)	24.0 (2.5)	0.001	−3.5 (−5.1 to −2.3)	0.024
31–40	127	32.9 (3.5)	33.8 (2.6)	0.002	0.9 (0.3 to −1.4)	0.241	14	27.5 (14.2)	33.3 (2.4)	0.196	5.7 (−3.3 to −14.9)	0.081
≥40	20	36.7 (2.9)	42.1 (1.6)	0.001	5.4 (4.0 to 6.8)	0.114	–	–	–	–	–	–
Weight status												
BMI < 25 (kg/m^2^)	348	28.5 (4.9)	24.5 (5.1)	0.001	−4.0 (−4.6 to −3.3)	0.051	307	23.4 (4.4)	13.3 (4.4)	0.001	−10.0 (−10.6 to −9.4)	0.087
25 ≤ BMI < 30 (kg/m^2^)	94	34.0 (2.4)	33.4 (3.7)	0.001	−5.5 (−1.5 to −0.4)	0.014	104	27.4 (5.9)	21.7 (4.1)	0.001	−5.6 (−7.1 to −4.1)	0.018
30 ≤ BMI < 35 (kg/m^2^)	27	37.9 (2.9)	40.4 (3.2)	0.001	−2.4 (0.8 to 4.1)	0.003	23	32.2 (4.3)	29.1 (6.7)	0.009	−3.1 (−5.5 to −0.8)	0.365

Data are expressed as mean (SD). * Levels of adiposity (20–30; 31–40 and ≥40 BF%) were classified according to the National Health and Nutrition Examination Survey (NHANES) (1999–2004) by BIA in Spanish population [21]. CI = confidence interval.

**Table 3:** Comparison of BAI in different trials.

Study	Sample	Age (Years)	Device	Agreement between Measurement Methods/Bias	Main Finding
Present study	903 apparently healthy and sub-sample with overweight/obese	Mean age 21.4 ± 3.3	Tetrapolar frequency	Bland–Altman plots Male bias 9.1%, Female bias 3.2%, Total bias 6.0%	Overall, BAI overestimating BF%, in overweight subjects the BAI overestimated BF%, and obese group the BAI underestimated BF% both genders.
Geliebter et al. [10]	19 pre-bariatric surgery clinically severe obese, non-diabetic females	Mean age 32.6 ± 7.7	Tetrapolar frequency	Bland–Altman plots Bias 2.2%	BAI underestimating BF%
Bernhard et al. [11]	240 patients with severe obesity	Mean age 44.1 ± 11.1	A single-frequency	Intraclass correlation 0.74; 95% confidence interval = 0.68–0.79	The two methods were similar according to the intraclass correlation
Ezeukwu et al. [12]	30 obese females	Mean age 22.8 ± 3.3	A single-frequency	Bland–Altman plots Bias 15.0%	BAI underestimating BF%
Lemacks et al. [36]	187 overweight/obese postmenopausal females	Mean age 55.8 ± 3.3	Dual-energy X-ray	Concordance correlation coefficient *ρ*c = 0.39	Poor agreement strength between Dual-energy X-ray (DEXA) BF% and BAI overestimating BF%
Vinknes et al. [35]	5193 middle-aged (47–49 years) and elderly (71–74 years) males and females	Mean range 47–72	Dual-energy X-ray	Bland–Altman plots Bias in subjects with lower BF% 6.0%, Bias in subjects with higher BF% 1.9%	BAI overestimated adiposity in subjects with lower BF% (particularly in males) and underestimated it in overweight and obese subjects

## Abbreviations

The following abbreviations are used in this manuscript:

BAI	Body adiposity index
BF%	Body fat percentage
BMI	Body mass index
CEMA	Centre of Studies in Physical Activity Measurements (In Spanish)
DEXA	Dual-energy X-ray absorptiometry
LMICs	Low-to-middle income countries
*ρc*	Lin’s concordance correlation coefficient
*R*^2^	Coefficient of determination
WC	Waist circumference
WHtR	Waist-to-height ratio
